# Redescription of *Arcifronsarcifrontalis* Ding & Yang, 1986 (Hemiptera, Fulgoromorpha, Delphacidae)

**DOI:** 10.3897/zookeys.825.21872

**Published:** 2019-02-27

**Authors:** Hong-Xing Li, Lin Yang, Xiang-Sheng Chen

**Affiliations:** 1 Institute of Entomology, Guizhou University, Guiyang, Guizhou, 550025, China Guizhou University Guiyang China; 2 The Provincial Special Key Laboratory for Development and Utilization of Insect Resources, Guizhou University, Guiyang, Guizhou, 550025, China Guizhou University Guiyang China

**Keywords:** Bamboo planthopper, distribution, female genitalia, Fulgoroidea, Homoptera, taxonomy

## Abstract

The male of *Arcifronsarcifrontalis* Ding & Yang, 1986 (Hemiptera, Fulgoroidea, Delphacidae, Tropidocephalini) is redescribed. The female genitalia of the species here, is described and illustrated for the first time. The geographic distribution of the species and images of adult habitus are provided.

## Introduction

The bamboo-feeding planthopper genus *Arcifrons* (Hemiptera, Fulgoromorpha, Delphacidae, Delphacinae, Tropidocephalini) (type species: *A.arcifrontalis* Ding & Yang, 1986), was established by Ding and Yang (1986). Until now, of only one described species in the genus, from China and with reported plant associations feed on bamboo (Ding and Yang 1986; Ding 2006). Members always collected on the genus *Phyllostachys* of bamboo in Yunnan Province, China (Ding 2006; this paper). This species was described and illustrated by the original authors, with the same illustrations recycled in Ding (2006). However, identification of species may be difficult because original species descriptions are inadequate in that many features are not evaluated and included, especially the male genitalia. So it is necessary to add other more valid characters.

Herein, based on specimens of *A.arcifrontalis* collected from Yunnan Province, China by Chen and Yang in 15 August 2015, Li, Luo and Yang in 18 August 2018, we review the species, the male is redescribed and of the female genitalia is described and illustrated for the first time. The geographic distribution and images of adult habitus are given.

## Material and methods

The morphological terminology and measurements follow Yang and Yang (1986) and the morphological terminology of female genitalia follows Bourgoin (1993). Body length was measured from apex of vertex to tip of tegmina. Dry male specimens were used for the description and illustrations. External morphology was observed under a stereoscopic microscope and characters were measured with an ocular micrometer. Color pictures for adult habitus were obtained by KEYENCE VHX-1000 system. Images of adult habitus were obtained by Canon Digital Camera EOS 5Ds. The genital segments of the examined specimens were macerated in 10% KOH and drawn from preparations in glycerin jelly using a Leica MZ 12.5 stereomicroscope. Illustrations were scanned with Canon CanoScan LiDE 200 and imported into Adobe Photoshop 6.0 for labeling and plate composition.

Specimens examined are deposited in the Institute of Entomology, Guizhou University, Guiyang, China (**IEGU**).

## Taxonomy

### Arcifrons

Taxon classificationAnimaliaHemipteraDelphacidae

Ding & Yang, 1986

Arcifrons : Ding and Yang 1986: 421; Ding 2006: 124.

#### Type species.

*Arcifronsarcifrontalis* Ding & Yang, 1986, by original designation.

#### Differential diagnosis.

**Figures 1–4.**
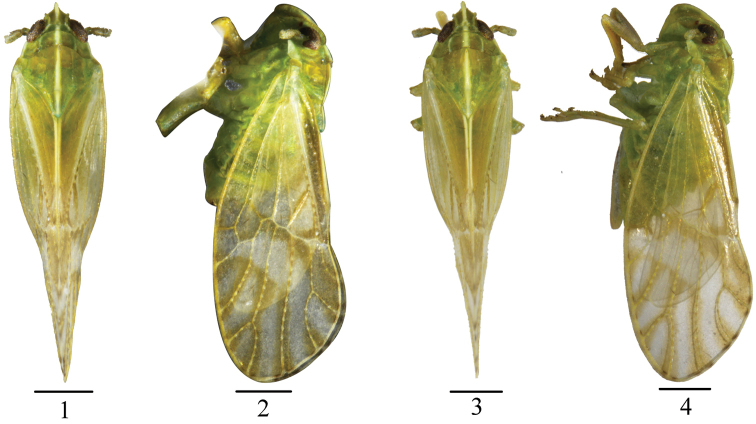
*Arcifronsarcifrontalis* Ding & Yang, 1986. **1** male habitus, dorsal view **2** the same, lateral view **3** female habitus, dorsal view **4** the same, lateral view. Scale bars: 0.5 mm.

**Figures 5–15.**
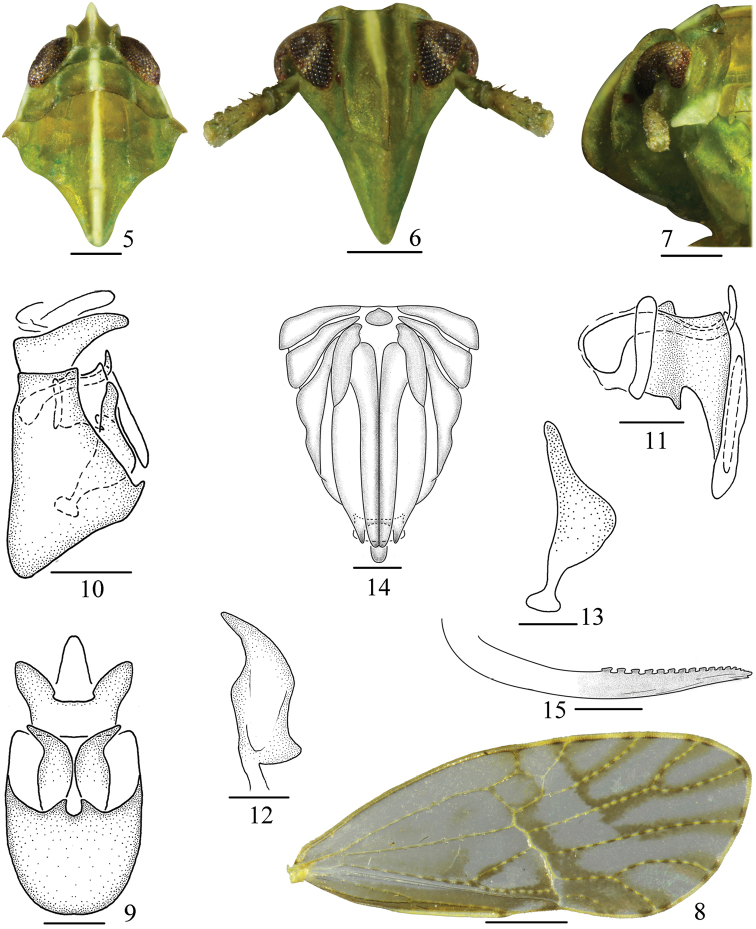
*Arcifronsarcifrontalis* Ding & Yang, 1986. **5** head and throad, dorsal view **6** frons and clypeus **7** the same, lateral view **8** tegmen **9** male genitalia, posterior view **10** the same, lateral view **11** aedeagus **12** genital style, posterior view **13** the same, left lateral view **14** female genitalia, posterior view **15** gonapophysis IX. Scale bars: 0.2 mm (**5–10, 14, 15**); 0.1 mm (**11–13**).

The genus is readily distinguished from other genera in the tribe Tropidocephalini by the following features: frons distinctly sharply pointed at apex in dorsal view (Figs 1, 3, 5) and distinctly inclined anteriorly in lateral view (Figs 2, 4, 7), with median carina distinctly developed (Fig. 6); postclypeus with median carina distinct (Fig. 6).

#### Description.

The distinctive characters used by Ding (2006) are modified as follows.

#### Head and thorax.

Head including eyes narrower than pronotum. Vertex broad transversely, with basal compartment near trapezoidal (Fig. 5). Frons elongate, rectangular, longer in middle line than wide at widest part, lateral carinae subparallel, median carina distinctly developed, forked at base (Fig. 6), with distinctly sharply pointed at apex in dorsal view (Fig. 5) and distinctly inclined anteriorly in lateral view, with apical margin roundly convex (Fig. 7). Postclypeus with median carina distinct, lateral carinae absent, width at base slightly wider than frons at apex. Antennae short, cylindrical, reaching to frontoclypeal suture, with basal segment with length longer than width, shorter than second segment (Fig. 6). Pronotum longer than vertex, tricarinae distinct, lateral carinae reaching hind margin, with base curved inward, with posterior apex more closed anterior apex of lateral carinae of mesonotum. Mesonotum developed, tricarinae distinct, median carina complete, reaching tip of scutellum, lateral carinae reaching hind margin (Fig. 5). Spinal formula of hind leg 5-6-4. Hind tibiae with a lateral tooth basally and medially respectively. Post-tibial spur without teeth along hind margin, but with a small apical tooth, with inner side surface concave.

#### Male genitalia.

Anal segment (Fig. 9) ring-like. Pygofer with ventral margin longer than dorsal margin in lateral view (Fig. 10), mediovental processes distinct (Fig. 9). Aedeagus (Fig. 11) with phallobase distinct. Genital styles (Figs 12, 13) simple, long.

#### Host plants.

Bamboo.

#### Distribution.

Oriental Region (China).

#### Remarks.

This genus is similar to *Arcofaciella* Fennah, 1956 but differs from it by: frons with median carina distinctly developed, with lateral carinae subparallel (frons with median carina not delveloped, with lateral carinae arched in *Arcofaciella*); postclypeus with median carina developed (postclypeus with median carina absent in *Arcofaciella*); spinal formula of hind leg 5-6-4 (spinal formula of hind leg 5-8-5 or 5-9-5 in *Arcofaciella*); anal segment of male with two processes large and lamellate (anal segment of male with two processes short and spinous in *Arcofaciella*).

This genus is also similar to *Mucillnata* Qin & Zhang, 2010 but differs from it by: frons distinctly inclined anteriorly in lateral view (frons not distinctly inclined anteriorly in lateral view in *Mucillnata*); lateral carinae of the pronotum not diverging and attaining the hind margin (lateral carinae of the pronotum diverging and not attaining the hind margin in *Mucillnata*); pygofer of male with ventral margin with two processes (pygofer of male with a medioventral process in *Mucillnata*); anal segment of male with two lateral processes (anal segment of male with single process on the caudoventral margin on right side in *Mucillnata*).

### Arcifrons arcifrontalis

Taxon classificationAnimaliaHemipteraDelphacidae

Ding & Yang, 1986

Figures 1–4 , 5–15 , 17 , 18

Arcifrons arcifrontalis : Ding and Yang 1986: 421; Ding 2006: 124.

#### Specimens examined.

17♂♂, 20♀♀, **China**: Yunnan, Yingjiang County (24°44N, 97°33E), on bamboo, 15 August 2015, Xiang-Sheng Chen and Lin Yang; 12♂♂, 18♀♀, Yunnan, Yingjiang County, on bamboo, 18 August 2018, Hong-Xing Li, Qiang Luo and Liang-Jing Yang.

#### Measurements.

Body length (from apex of vertex to tip of tegmina): male 3.2–3.5 mm (*N* = 17); female 3.4–3.8 mm (*N* = 20); tegmen length: male 2.7–3.0 mm (*N* = 17); female 2.8–3.3 mm (*N* = 20).

#### Coloration.

General color yellowish green (Figs 1–4). In dorsal view, a white large longitudinal stripe along median line from basal half of frons to the end of mesonotum (Fig. 5). Rostrum with apex dark brown. Eyes yellowish brown to blackish brown. Ocelli reddish brown. Pronotum with a white longitudinal band along lateral margin (Fig. 5). Tegulae yellowish white. Tegmina with white spots along longitudinal veins in apical half, along transverse vein and apical veins bordered yellowish brown stripes as figured (Fig. 8). Wings hyaline, with veins brown.

#### Head and thorax.

Ratio width of vertex at base to length 2.9–3.2, to width at apex 1.3–1.5, lateral margin slightly keeled, Y-shaped carina distinct (Fig. 5). Frons with ratio length to width 1.5–1.8, the widest at near ocelli. Ratio length of rostrum to width 7.4–7.6. Basal segment of antennae with ratio length to width 1.3–1.6, to length of second segment 0.5–0.6 (Fig. 6). Pronotum with ratio length in midline to length of vertex 2.2–2.4, anterior margin straight, posterior margin concave. Mesonotum in midline 3.5–3.8 times longer than pronotum, 2.4–2.7 times longer than vertex and pronotum combined (Fig. 5). Tegmina (Fig. 8) amply exceeding the tip of abdomen, 2.5 times longer than wide, with apical margin broadly rounded; Sc+R and M with common petiole at base, and forked before midline; Sc and R with two branches respectively; M with three branches at apical, M_1_ fused with Rs basally, and M_3_ fused with Cu_1_a basally; Cu with three branches; A with two branches fused at apical half.

#### Male genitalia.

Anal segment short, with inverse collar-shaped in posterior view, with lateral processes large and lamellate, apical margin roundly convex (Fig. 9). Pygofer with mediovental processes paired, finger-like, directed each other, opening oval, longer than wide, with ratio length to width 1.5 (Fig. 9), in profile with dorsal margin longer than ventral margin (Fig. 10). Aedeagus (Fig. 11) with phallobase distinct. Phallus tubular, with basal third exceeding the phallobase cephalad, and with apical fifth exceeding phallobase caudad, directed dorsad. Phallobase with apical two thirds narrowing apically, directed ventrad, dorsal margin with a stout tooth-like process at base and ventral margin with another small tooth-like process at basal third, in posterior view ring-like, opening narrow. Genital styles in profile (Fig. 13) with ventral margin distinctly convex, and with dorsal margin slightly concave, in posterior view (Fig. 12) moderately long, flake-shaped, broad at middle, apex acute, reaching the base of anal segment.

#### Female genitalia.

Anal style exceeded pygofer. Pygofer with gonocoxa VIII moderately large, basal third with inner lateral margin sinuate. Ovipositor longer than pygofer. Gonangulum small, with width wider than length, basal margin subangular convex in the middle, apical margin slightly convex, separated from gonocoxa VIII. Gonoplacs elongate, sword-like, with apex beyond apical margin of pygofer (Fig. 14). Gonapophyses IX (Fig. 15) slender, gradually narrowed apically, apex sharp, dorsal margin with apical half serrated.

#### Host plant.

Bamboo (*Phyllostachys* sp.).

#### Distribution.

**Figure 16.**
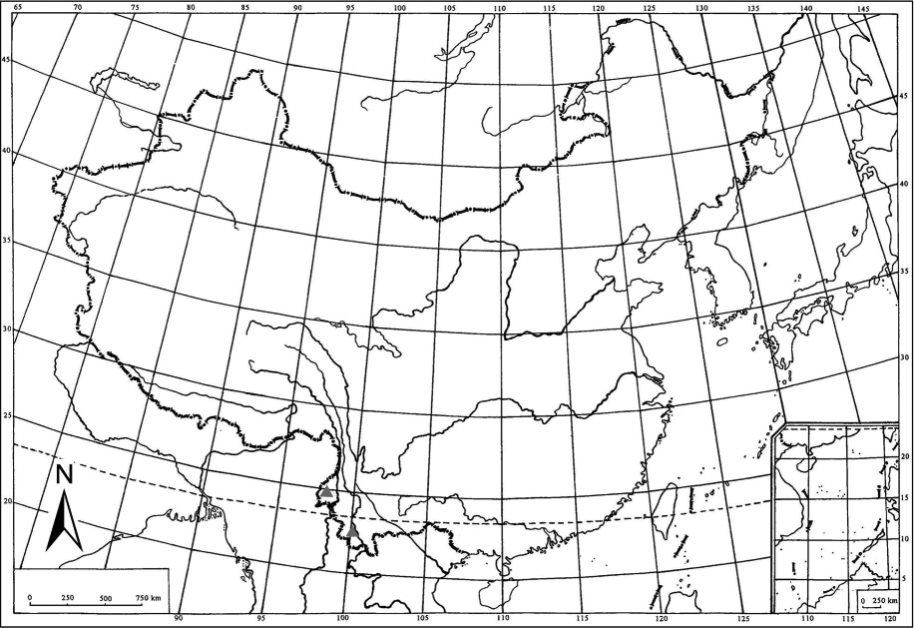
Geographic distribution of *Arcifronsarcifrontalis* Ding & Yang, 1986 in China (black triangle).

Southwest China (Yunnan Province) (Fig. 16).

**Figures 17, 18.**
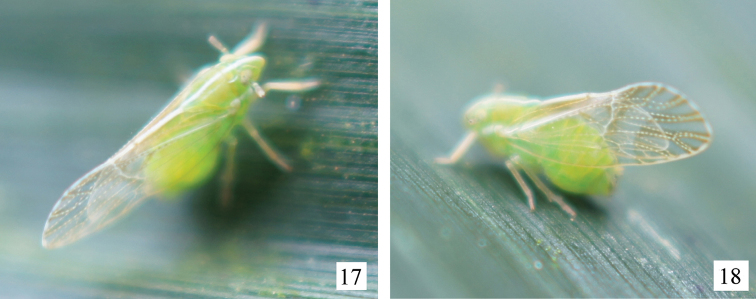
Adult of *Arcifronsarcifrontalis* Ding & Yang, 1986 resting on leaf of bamboo. Photographed by Xiang-Sheng Chen.

## Discussion

The discovery of the species broadens our knowledge of the morphology and biogeography of the genus. Species of *Arcifrons* feed exclusively on bamboo and occur in Yunnan, China. This may be due to the climate warm and humid, subtropical monsoon climate of Yunnan, with minimal temperature changes. Members are collected on leaves of the genus *Phyllostachys* of bamboo. The genus *Phyllostachys*, with at least 51 species, has the highest species density in China (49 species). Many of the species are found in central and southern China (Wang and Stapleton 2006). Therefore, species of *Arcifrons* may be more widely distributed in China than hitherto reported, and that there may be many undescribed species in this genus. *Arcifronsarcifrontalis* Ding & Yang is of economic significance since the species has large population in the bamboo fields.

## Supplementary Material

XML Treatment for Arcifrons

XML Treatment for Arcifrons arcifrontalis
